# Clinical use, diagnostic efficiency and impact on patient management of cardiovascular magnetic resonance

**DOI:** 10.1186/1532-429X-14-S1-P41

**Published:** 2012-02-01

**Authors:** Florian von Knobelsdorff-Brenkenhoff, Angelika Bublak, Sana El-Mahmoud, Ralf Wassmuth, Christian Opitz, Jeanette Schulz-Menger

**Affiliations:** 1 Working Group Cardiovascular MRI, Charite Medical University Berlin, Berlin, Germany; 2 Cardiology and Nephrology, HELIOS Klinikum Buch, Berlin, Germany; 3 Cardiology, DRK Klinikum Berlin-Koepenick, Berlin, Germany; 4 Internal Medicine B, Medical University, Greifswald, Germany

## Background

To evaluate the clinical use, diagnostic efficiency and impact on patient management of cardiovascular magnetic resonance (CMR).

## Methods

Data of the individual patient as well as procedural information of 2598 consecutive clinically indicated CMR exams were prospectively collected in a single CMR center. For a representative subgroup of 250 exams, an external blinded reviewer evaluated the need for diagnostic tests and hospitalization before and after the CMR exam and assessed the impact of CMR on patient management.

## Results

CMR was used in a large variety of indications, with inflammatory (29.4%) and ischemic (26.2%) heart disease as the most frequent. All moderate adverse events (0.5%) were associated with stress medication or contrast media. In subgroup analysis, CMR ruled out a suspected disease in 63.1%, confirmed a suspected or known disease in 27.8% or detected an unexpected new pathology in 7.2%. The clinical question was answered completely or partially in 88.4% and 11.2%, respectively. CMR reduced the amount of other diagnostic tests by 65.7% and changed the diagnostic strategy for the individual patient in 92.4%. For instance, 11/36 (30.6%) of the subjects who should initially undergo heart catheterization did not require this invasive study subsequent to the CMR. In contrast, 12/214 (5.6%) of the patients required heart catheterization based on the CMR findings, even though they were not scheduled for heart catheterization initially. In 23.6%, CMR led to a strategic change between in- or outpatient treatment.

## Conclusions

CMR is safe, robust and versatile. It influences the indication for hospital admission, the choice and extent of individual downstream diagnostic testing, and the determination of the final diagnosis.

## Funding

The study is supported by a grant of the HELIOS Research Center, Berlin, Germany.

